# Current Surgical Management for Acral Melanoma

**DOI:** 10.1007/s11864-025-01361-1

**Published:** 2025-10-27

**Authors:** Shigeru Koizumi, Takashi Inozume, Yasuhiro Nakamura

**Affiliations:** 1 Department of Skin Oncology/Dermatology, Saitama Medical University International Medical Center, 1397-1 Yamane, Hidaka, Saitama Japan; 2 Department of Dermatology, Chiba University, 1-8-1 Inohana, Chuo-ku, Chiba, Japan

**Keywords:** Acral melanoma, Complete lymph node dissection, Deep margin, Mohs surgery, Sentinel lymph node biopsy, Peripheral margin

## Abstract

Melanoma is one of the most aggressive and lethal forms of skin cancer, with acral melanoma (AM) associated with the poorer prognosis among melanoma subtypes. Historically, it was considered that more extensive surgery could prolong survival; however, multiple randomized trials have demonstrated that greater surgical intervention does not improve survival. Over the past few years, novel therapeutic agents including immune checkpoint inhibitors and molecular-targeted drugs have remarkably improved prognosis of melanoma and potentially reduced the role of surgery. Furthermore, predictive models that integrate clinicopathologic features and gene expression profiling may further optimize patient selection and guidance for surgical de-escalation. In parallel, the use of these agents in adjuvant and neoadjuvant settings highlights the need for multimodal approaches combined with surgery. However, the landmark clinical trials have included few cases of AM, which is rare melanoma subtype in Western populations. Because of its unique molecular alterations, the applicability of findings from Western-based clinical trials to AM remains uncertain, leading to a lack of high-level evidence for this subtype. In this article, we review the available, albeit limited, evidence on surgical management for AM and discuss future perspectives and challenges for optimizing treatment strategies for this distinct melanoma subtype.

## Introduction

Before the invention of novel therapeutic agents such as immune checkpoint inhibitors (ICIs) and molecular targeted drugs, surgery was one of the few available and effective treatment options [1–9]. Historically, radical clearance of primary lesion with larger margin, complete lymph node dissection (CLND), and metastasectomy could improve prognosis [1, 10]. However, multiple international clinical trials regarding peripheral margin and lymph node surgery have demonstrated that aggressive surgical procedure could not lead to survival benefit [11–23]. While surgery remains the standard of care for localized melanoma, the advent of novel anti-tumor agents and the increasing implementation of multimodal treatment strategies have led to a shift toward less invasive surgical approaches [10, 24, 25].

Notably, the acral melanoma (AM) is a rare subtype of melanoma in the Caucasian population and differs from other subtypes such as non-acral cutaneous melanoma (NACM) for genetic mutations and biological behavior [26–28]. Due to its rarity, few patients with AM were included in the landmark trials [11–23]. Furthermore, the palm, sole, and nail apparatus, where AM predominantly arises, are anatomically complex and play a crucial role for daily functional activities. Extensive surgical deficit leads to not only post operative morbidity but also a decreased quality of life (QOL) [29]. Accordingly, surgical management should be carefully tailored to balance oncologic control with functional preservation. Herein, we provide an overview of the latest evidence and discuss future directions in surgical management for AM.

## Characteristics of Acral Melanoma

AM is a clinically distinct subtype, characterized by unique epidemiological, anatomical, and genomic features that set it apart from NACM. The incidence of AM differs markedly across ethnic and geographic populations [30–33]. In European countries and North America, AM represents only about 0.8–3% of all melanoma cases [31, 33]. In contrast, its prevalence is strikingly higher in Asian countries, accounting for approximately 40–58% of all melanoma cases [30, 32].

AM typically arises on the palm, sole, and nail apparatus, which are generally shielded from chronic ultraviolet (UV) exposure. This contrasts with NACM, whose predominant pathogenesis is UV-induced damage. In the sole AM, mechanical stress associated with weight-bearing has been implicated as a potential driver of tumor genesis [34, 35]. Such external stress has been shown to induce nuclear membrane disruption and genomic instability through YAP activation, leading to UV-independent mutations [35]. Consistent with its low UV exposure and alternative pathogenic mechanisms, AM generally exhibits a lower tumor mutational burden than NACM [26]. In addition, AM is characterized by a higher frequency of DNA structural variants, further supporting the notion that its genomic landscape reflects a distinct pathogenesis from that of NACM [26, 36–39]. Moreover, driver mutation is also different between subtypes. While mutations in *C-KIT*, *CDK4*,* CCND1*,* and CDKN2A* are frequently observed in AM, NACM more commonly harbors driver mutations in *BRAF*, *CDKN2A*, *NRAS*, and *TP53* [26, 40]. Although *BRAF* mutations are found in both subtypes, their prevalence is considerably lower in AM (less than 10%) compared with in NACM (40–60%) [26, 41]. Consequently, only a minority of AM patients are eligible for BRAF/MEK therapy.

Regarding the tumor microenvironment, AM exhibits a characteristic profile of tumor infiltrating lymphocyte. The regulatory T cells are more abundant in AM than in NACM [42, 43]. In contrast, AM showed a lower frequency of tumor-reactive CD8 + T cells compared with NACM [43]. These findings indicate that AM is characterized by an immunosuppressive tumor microenvironment, resulting in lower tumor response to ICIs relative to NACM [37–39, 44–46]. Accordingly, AM have reported to be poorer prognosis than those with NACM [32, 37, 39].

## Wide Local Excision of Primary Lesion

### Peripheral Margins

Adequate peripheral margins are essential to achieve complete tumor clearance, including the removal of microscopic satellite metastases and occult horizontal spread [14–17, 19, 47–49]. Historically, wider peripheral margins were thought to improve local control and overall survival, leading to more aggressive surgical strategies for primary lesions; wide local excision (WLE) with a 5 cm peripheral margin was commonly performed [1]. Thereafter, to determine an appropriate margin that minimizes morbidity without compromising survival, six pivotal international randomized trials comparing different peripheral margins have been conducted (Table 1) [14–17, 19, 47, 48]. Based on these trials, the current world guidelines, such as the National Comprehensive Cancer Network (NCCN) Guidelines, European Society for Medical Oncology Clinical Practice Guidelines, and European Association of Dermato-Oncology Guidelines, and Japanese Dermatological Association Guidelines, recommend peripheral margins according to Breslow thickness (BT) [25, 50–53].

**Table 1:** Clinical trials evaluating peripheral margins in melanoma

Author^reference no^.	Published year	Total sample size	Median follow-up period (years)	Breslow thickness (mm)	Peripheral margin (cm)	Local recurrence	Overall survival	Sample size of acral melanoma
[11, 12]	1991	612	7.5	≤ 2.0	1 vs. ≥3	Not significant	Not significant	Not specified
[14]	2000	989	11	0.8–2.0	2 vs. 5	Not significant	Not significant	6
[15]	2001	468	10	1.0–4.0	2 vs. 4	Not significant	Not significant	Not specified
[16]	2003	326	16	≤ 2.0	2 vs. 5	Not significant	Not significant	0
[19]	2011	936	6.7	> 2.0	2 vs. 4	Not significant	Not significant	2
[17]	2004	900	8.8	>2.0	1 vs. 3	Significant^†^	Not significant^‡^	0
[48]	2016							

^†^Including local recurrence, in- transit metastasis, and regional lymph node metastasis

^‡^Statistically significant in melanoma- specific survival

However, these trials were primarily conducted in Caucasian populations, in whom melanoma commonly arises on the trunk or extremities. As a result, few patients with AM were included due to its rarity in these cohorts (Table 1). Consequently, the optimal peripheral margin for AM has not been adequately investigated in randomized studies, and current evidence relies largely on a limited number of retrospective analyses (Table 2) [54–57].

**Table 2:** Retrospective studies on peripheral margins in acral melanoma

Author ^reference no^.	Peripheral margin (cm)	Total sample size	T stage	Disease-free survival (DFS)		Overall survival (OS)		Melanoma-specific survival (MSS)	
				5-year DFS	Multivariate analysis	5-year OS	Multivariate analysis	5-year MSS	Multivariate analysis
[54]	Narrow vs. recommended	100	pT1–T4	57.1% vs. 81.1% *P* = 0.02	HR, 1.73 *P* = 0.31	N/A	N/A	71.3% vs. 87.5% *P* = 0.045	HR, 1.83 *P* = 0.38
[55]	1–2 vs. ≥2	207	pT3–T4	N/A *P* = 0.08	HR, 1.09 *P* = 0.76	N/A *P* = 0.20	HR, 1.07 *P* = 0.87	N/A	N/A
[56]	0.5 vs. 1	39	pT1a	N/A	N/A	N/A	N/A	N/A *P* = 0.40^†^	N/A
[58]	1 vs. 2	336	pT3–T4	33.9% vs. 39.9% *P* = 0.35	HR, 1.05 *P* = 0.75	60.7% vs. 60.3% *P* = 0.67	HR, 1.20 *P* = 0.36	66.7% vs. 68.4% *P* = 0.36	HR, 1.05 *P* = 0.82

^†^Crude cumulative incidence was used to analyze the data

*HR* hazard ratio, *N/A* not available

Ito et al. retrospectively analyzed 100 Japanese patients with pT1–T4 AM to compare clinical outcomes between two surgical strategies: one using the NCCN Guidelines-recommended margins and another with narrower margins [54]. Kaplan–Meier analysis indicated that both disease-free survival (DFS) and melanoma-specific survival (MSS) were significantly lower in the group treated with guideline-recommended margins than the narrower-margin group (5-year DFS: 57.1% vs. 81.1%, *P* = 0.02; 5-year MSS: 71.3% vs. 87.5%, *P* = 0.045). After adjustment for confounding factors using a multivariate Cox proportional hazards model, peripheral margin was not significantly associated with DFS or MSS (DFS: hazard ratio [HR], 1.73; *P* = 0.31; MSS: HR, 1.83; *P* = 0.38).

Similarly, Sun et al. retrospectively evaluated the impact of different peripheral margins (1–2 cm vs. ≥2 cm) on local/in-transit recurrence-free survival (LITRFS), DFS, and overall survival (OS) in 207 patients with pT3–T4 AM [55]. The Kaplan–Meier analysis showed no significant differences in LITRFS, DFS, or OS between the two cohorts (*P* = 0.35, 0.08, and 0.20). Multivariate Cox proportional hazards model confirmed that a narrower margin (1–2 cm) was not associated with adverse outcome (DFS: HR, 1.09; *P* = 0.76; OS: HR, 1.07; *P* = 0.87).

In another study, Maurichi et al. retrospectively compared survival in 39 patients with pT1a AM who underwent WLE with either a 0.5–1 cm peripheral margin [56]. The crude cumulative incidence of local recurrence and melanoma-specific death was similar between the two groups (*P* = 0.45; *P* = 0.40).

Finally, Koizumi et al. conducted a multi-institutional retrospective study including 336 Japanese patients with pT3–T4 AM of the sole, comparing 1 cm vs. 2 cm peripheral margins [58]. No significant differences were observed between the two groups in local recurrence-free survival (LRFS), DFS, OS, or MSS (5-year LRFS: 58.1% vs. 58.3%, *P* = 0.84; 5-year DFS: 33.9% vs. 39.9%, *P* = 0.35; 5-year OS: 60.7% vs. 60.3%, *P* = 0.67; 5-year MSS: 66.7% vs. 68.4%, *P* = 0.36). Multivariate Cox proportional hazards model also showed no negative impact of the 1 cm margin on survival outcomes (LRFS: HR, 1.19; *P* = 0.38; DFS: HR, 1.05; *P* = 0.75; OS: HR, 1.20; *P* = 0.36; MSS: HR, 1.05; *P* = 0.82).

Collectively, these studies suggest that narrower surgical margins, compared to the guideline recommendations, may not compromise oncologic outcomes in AM. Given the anatomical and functional constraints of typical AM sites, narrower margins could be considered to reduce morbidity while preserving function. However, because current evidence is derived from retrospective studies, prospective trials are needed to establish optimal margin recommendations for this rare subtype.

### Deep Margins

Taking an adequate deep surgical margin is essential to ensure complete tumor removal, eliminate microscopic tumor spread, and maintain effective local control. The NCCN Guidelines recommend excising all tissue above the fascia during WLE for invasive melanoma, and resecting the fascia itself if it is involved [50]. However, robust evidence defining the optimal depth of WLE is lacking, as no prospective studies have addressed this issue [59–62]. To date, only a few retrospective studies have specifically focused on deep margin of WLE in AM [63].

**Fig. 1:**
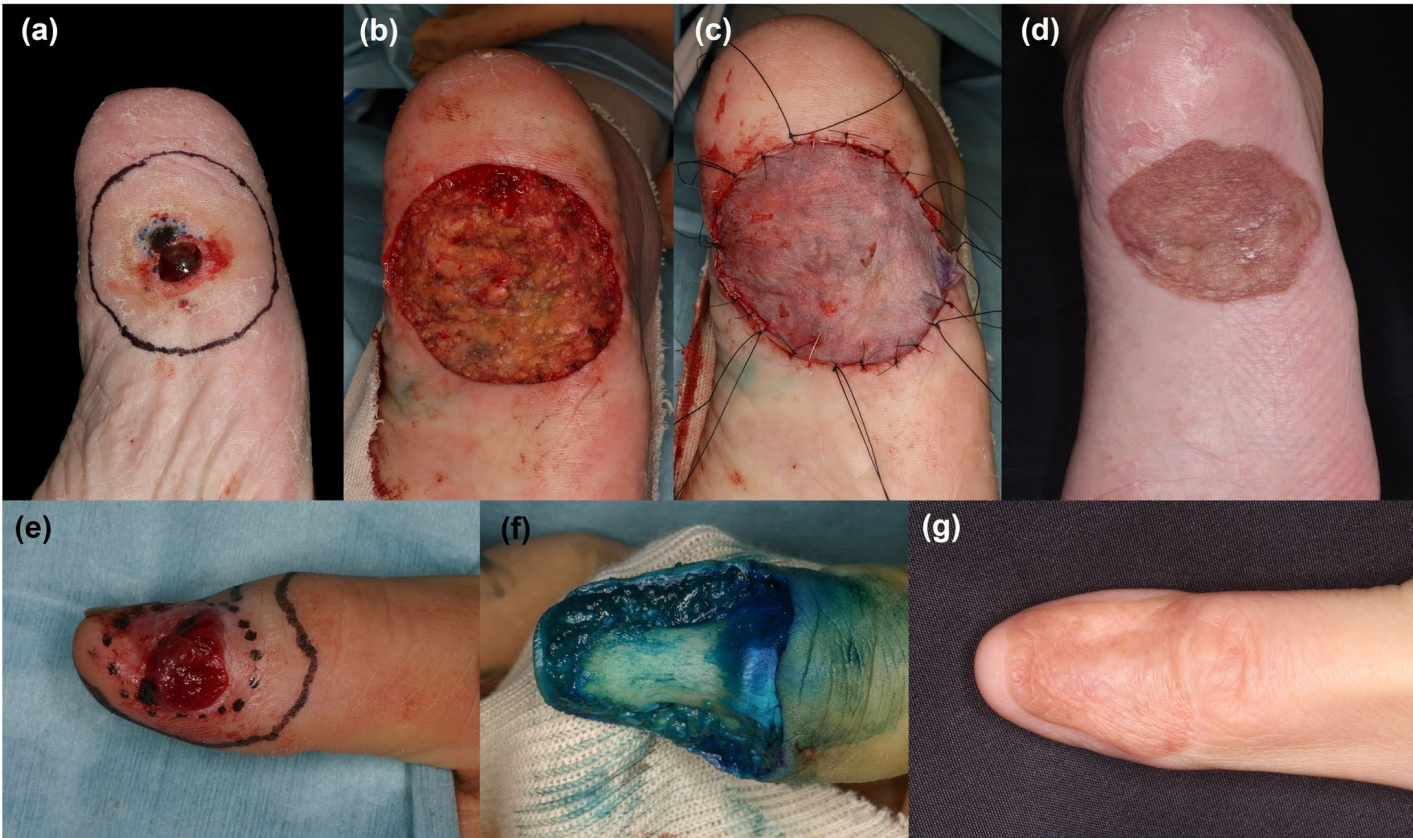
Representative surgical cases of acral melanoma of the sole and nail apparatus. (**a**) Invasive acral melanoma of the heel with a Breslow thickness of 2.8 mm. (**b**) The tumor was excised at the level of the superficial subcutaneous fat, preserving the fat pad. (**c**) The defect was reconstructed with a split thickness skin grafting. (**d**) Clinical appearance at 4 months after skin grafting. (**e**) Invasive nail apparatus melanoma of the thumbnail with a Breslow thickness of 5.2 mm. (**f**) The tumor was excised including the periosteum while preserving the distal phalanx. (**g**) Clinical appearance at 7 years after full-thickness skin grafting

Koizumi et al. retrospectively evaluated the prognostic impact of different deep margin depths (within vs. beyond the subcutaneous fat) in 464 Japanese patients with invasive AM of the sole who underwent WLE (Fig. 1a-d) [63]. After propensity score matching (*n* = 139 per cohort), survival outcomes were comparable between the two groups (5-year LRFS: 72.8% vs. 66.8%, *P* = 0.55; 5-year DFS: 55.3% vs. 43.7%, *P* = 0.24; 5-year OS: 76.2% vs. 73.2%, *P* = 0.52).

The nail apparatus is anatomically distinct from other cutaneous sites, characterized by the absence of a well-defined fascial layer and minimal distance to the periosteum [64, 65]. These characteristics necessitate a tailored surgical approach. Oh et al. retrospectively investigated recurrence risk in 140 Korean patients with nail apparatus melanoma (NAM), comparing digit-sparing surgery with amputation, with median follow-up periods of 3.8 and 4.0 years, respectively [66]. Multivariable Cox proportional hazards model showed that digit-sparing surgery was not significantly associated with increased risk of recurrence (adjusted HR [aHR], 1.67, 95% confidence interval [CI]: 0.74–3.77) or distant metastasis (aHR, 0.45, 95% CI, 0.16–1.28). Moreover, receiver operating characteristic analysis identified 0.8 mm as the optimal cut-off for predicting recurrence, with a negative predictive value of 88%. These findings support digit-sparing surgery as a feasible option for NAM with a BT < 0.8 mm, in line with NCCN Guidelines, which recommend its consideration for in situ and selected minimally invasive tumors following adequate biopsy confirmation [50]. Furthermore, an investigator-initiated single-arm trial, JCOG1602 (J-NAIL study, clinical trial no. UMIN000029997), is currently ongoing in Japan to evaluate the efficacy and safety of digit-sparing surgery in patients with invasive NAM (Fig. 1e-g) [67].

## Mohs Micrographic Surgery

Mohs micrographic surgery (MMS) offers the distinct advantage of comprehensive margin control while maximizing tissue preservation by excising additional tissue only where histologically positive margins are identified [68, 69] However, MMS has several limitations, including the suboptimal diagnostic reliability of frozen sections for certain melanoma subtypes, longer procedural duration, and higher costs [68, 69]. Notably, no randomized trials have directly compared MMS with conventional WLE for melanoma, and robust evidence regarding its oncologic efficacy and safety remains lacking [68–73]. Reflecting these uncertainties, the current NCCN Guidelines do not recommend MMS for primary invasive melanoma when an adequate peripheral margin can be achieved through standard excision [50]. However, MMS may be considered in selected cases of minimally invasive (pT1a) melanoma, whose risk of recurrence is low, located in anatomically sensitive regions, such as the face, ears, and acral sites, where the preservation of function and cosmesis is paramount [50].

To date, only two retrospective studies have examined MSS for AM [72, 73]. Seo et al. compared slow MMS, a modified approach using formalin-fixed, paraffin-embedded sections for precise histopathological margin assessment, with WLE in 166 Korean patients with AM [72]. Unlike conventional MMS, slow MMS avoids reliance on frozen sections but requires several days for results. Multivariate Cox proportional hazards model demonstrated comparable recurrence-free survival (RFS) and OS between slow MMS and WLE (aHR, 0.77, *P* = 0.44; aHR, 0.89, *P* = 0.81). Importantly, the final surgical defect relative to tumor size was significantly smaller with slow MMS than with WLE (defect-to-tumor size ratio: 2.92 vs. 4.99, *P* < 0.001), suggesting superior tissue conservation. These findings indicate that slow MMS can achieve oncologic outcomes comparable to WLE while minimizing the extent of excision.

Similarly, Morales et al. evaluated MMS in 69 patients with NAM [73]. During a mean follow-up of 3.1 years, a high local control rate was obtained, with only one local recurrence (1.4%). Although only a small proportion of patients (7.2%) initially required amputation for tumor clearance, nearly half (47.8%) ultimately underwent amputation for reconstructive purposes due to extensive bone exposure. This highlights that while MMS provides excellent local control for NAM, its utility may be limited by the frequent need for secondary amputation. Both studies are limited by the inherent biases of retrospective designs, including potential biases related to patient selection and tumor characteristics.

Those findings does not lead to broader application of MMS due to the lack of comparative data. Further prospective studies are needed to better define the indications, safety, and long-term outcomes of MMS for AM.

## Lymph Node Surgery

### Sentinel Lymph Node Biopsy

Sentinel lymph node biopsy (SLNB) is a standard surgical procedure used to detect subclinical involvement of regional lymph nodes, providing critical information for staging, prognosis, and optimal management.

#### SNLB Versus Observation

The Multicenter Selective Lymphadenectomy Trial I (MSLT-I) demonstrated the clinical benefit of SLNB for patients with intermediate-thickness (1.2–3.5 mm) melanoma. Patients with positive SLNs who underwent immediate CLND showed significantly longer DFS than those who did not undergo SLNB and received delayed CLND at the time of nodal metastasis (*P* = 0.009). These findings underscore the survival benefit conferred by early detection of occult nodal metastases and prompt therapeutic intervention, highlighting the importance of selecting patients more likely to benefit from SLNB. Because the likelihood of positive SLNs is extremely low in patients with a BT < 0.8 mm without ulceration [18, 74–78]. the current NCCN Guidelines recommend discussing SLNB for patients with BT < 0.8 mm with ulceration or 0.8–1 mm regardless of ulceration, and offering it to those with a BT >1.0 mm [50].

Due to the lack of prospective trials for AM and the unknown proportion of AM cases in the MSLT-I, evidence on the impact of SLNB in AM is limited. In a retrospective study of 114 Taiwanese patients with AM, Chan et al. reported significantly longer DFS in the SLNB group than in the nodal observation group (median DFS: 62.6 vs. 19.3 months, *P* < 0.01), with a trend toward improved MSS (median MSS: not reached vs. 45.2 months, *P* = 0.05) [79]. Similarly, Farooq MS et al. analyzed 1,764 AM patients with BT ≥ 0.8 mm or ulceration using the National Cancer Database [80]. SLNB was associated with improved OS in multivariate Cox proportional hazards model (HR, 0.73, *P* = 0.02), and after the propensity score matching, the SLNB group showed significantly longer OS than the non-SLNB group (5-year OS: 75% vs. 62%, *P* = 0.04). Collectively, these retrospective studies suggest that SLNB may provide a prognostic benefit in AM.

#### Predictive Models for SLN Metastases

The recent development of predictive models for SLN metastasis based on combined clinicopathologic (CP) features and gene expression profiling (GEP), is reshaping the role of SLNB. Bellomo et al. proposed a CP–GEP model integrating variables such as Breslow thickness and age with a GEP signature including melanosome-related genes (*MLANA*) and epithelial–mesenchymal transition genes (*GDF15*, *CXCL8*, *LOXL4*, *TGFBR1*, *ITGB3*, *PLAT*, and *SERPINE2*) [81]. Derived from diagnostic biopsy tissue, the model predicts SLN metastasis risk before WLE ± SLNB. In a validation cohort of 754 patients (AM: *n* = 17; BT 0.5–4.0 mm), the model achieved a negative predictive value (NPV) of 96% and reduced SLNB procedures by 42% [81]. Amaral et al. further demonstrated its prognostic value [82]. In 543 stage I/II melanoma patients (AM: *n* = 72) who had negative SLN, the 5-year RFS was significantly higher in the low-risk than the high-risk group (77.8% vs. 93.0%, *P* < 0.001) [82]. A systematic review and meta-analysis showed pooled sensitivity, NPV, and SLNB reduction rates of 93%, 95%, and 27% in T1–T4 melanoma, with similarly high NPV in T2 melanoma (96%) and a 31% reduction rate [83]. This model has also shown utility in patients who did not undergo SLNB [84]. Among 930 clinically staged I/II melanoma patients (AM: *n* = 43), high-risk classification was associated with markedly worse RFS and MSS than in the low-risk cohort (10-year RFS: 37.5% vs. 94.5%, *P* < 0.001; 10-year MSS: 66.8% vs. 98.6%, *P* < 0.001). Subgroup analyses in AM showed similar trends.

These results suggest that the CP–GEP model may help identify patients, including AM, at low risk who could forgo SLNB, and provide prognostic information even with negative or unknown SLN status. Nevertheless, SLNB remains indispensable for confirming nodal status, ensuring accurate pathological staging, and guiding adjuvant therapy. CP–GEP model should be considered a complement rather than a replacement.

### Complete Lymph Node Dissection

CLND in melanoma is primarily indicated for patients with a positive SLN or clinical nodal metastases [20].

#### CLND for Patients with SLN Metastases

Historically standard after a positive SLN, its role has shifted following phase III randomized trials (MSLT-II and DeCOG-SLT), which showed no survival benefit for immediate CLND compared with observation and delayed CLND upon clinical progression [21, 22, 85, 86]. Consequently, omission of CLND in positive SLN patients has increased in a real-world practice [85, 87]. However, none of these trials included AM patients, and whether the results apply to this subtype, characterized by high SLN positivity, limited response to immunotherapy, and poorer prognosis, remains unclear [38, 88, 89].

Retrospective studies in AM have yielded conflicting results: in the Taiwanese study, immediate CLND (*n* = 27) improved DMFS versus observation (*n* = 6) (2-year DMFS: 63.0% vs. 50.0%, *P* = 0.046) [90], whereas in the Chinese study, no survival benefit was seen between the immediate CLND (*n* = 64) and observation (*n* = 21) groups (RFS: *P* = 0.52) [91]. Notably, both studies lacked sufficient report of patient background, warranting caution in interpreting their findings. These discrepancies highlight the need for larger, well-designed studies to clarify the role of CLND in AM patients with a positive SLN.

#### CLND for Patients with Clinical Nodal Metastases

For patients with clinical nodal metastases, CLND remains a standard of care [50]. However, its role is evolving with neoadjuvant therapy.

One important aspect is the potential for neoadjuvant therapy, when added to surgery, to offer superior survival outcomes compared with CLND, followed by adjuvant therapy alone. In a phase II randomized trial (S1801) of patients with stage IIIB–IIID melanoma with clinical nodal metastases or resectable stage IV melanoma, event-free survival (EFS) was significantly higher in the neoadjuvant–adjuvant pembrolizumab group than in the adjuvant pembrolizumab-only group (2-year EFS: 72% vs. 49%, *P* = 0.004) [92]. Notably, only nine AM patients were enrolled, and two in the adjuvant-only group died [92]. In addition, a phase III randomized trial (NADINA) in stage IIIB–IIID melanoma with clinical nodal metastases demonstrated significantly higher EFS in the neoadjuvant ipilimumab plus nivolumab arm than in the adjuvant nivolumab arm (1-year EFS: 83.7% vs. 57.2%, *P* < 0.001) [93]. However, the number of AM patients enrolled in this trial was not reported.

Another important aspect is the potential for neoadjuvant therapy to permit omission of CLND. In the phase II clinical trials of OpACIN-neo [94] and PRADO [95], stage IIIB–IIID melanoma patients with clinical nodal metastases received neoadjuvant ipilimumab plus nivolumab, followed either by CLND [94] or, in patients achieving a major pathologic response (MPR; ≤10% viable tumor) in the index node, by omission of CLND [95]. A post-hoc pooled analyses of the two trials demonstrated that omission of CLND did not adversely affect RFS in patients achieving an MPR (log rank test: *P* = 0.47; Cox multivariate hazard model: HR, 1.84, *P* = 0.48) [96]. Again, the number of AM patients included in these trials was not specified.

As for the prospective studies specifically targeting AM, only one phase II single-arm trial (CAP 03-Neo) has evaluated the efficacy of neoadjuvant camrelizumab plus apatinib, and temozolomide in stage II/III AM [97]. Among stage III patients (*n* = 17), the MPR rate was 52.9% and the 1-year EFS of 75.6% [97]. Nevertheless, no definitive consensus exists on integrating neoadjuvant therapy and CLND for AM. Future advances in neoadjuvant therapy may enable more personalized management of this subtype, potentially reducing the need for CLND, a highly invasive surgical procedure.

## Conclusions

To date, multiple randomized trials on surgical procedures have shown that less invasive approaches do not compromise survival. However, these trials have included very few AM cases, resulting in limited evidence for this subtype. Although retrospective studies on surgical treatment for AM are few, their findings generally align with those of the randomized trials. With the advent of novel therapeutic agents, integrating these treatments with predictive models may facilitate the use of less invasive surgical approaches.

## Key References

- Reijers ILM. et al. Impact of personalized response-directed surgery and adjuvant therapy on survival after neoadjuvant immunotherapy in stage III melanoma: Comparison of 3-year data from PRADO and OpACIN-neo. Eur J Cancer. 2025;214:115141.
  - This reference is of outstanding importance because it provides post-hoc analysis of two neoadjuvant trials, PRADO and OpACIN-neo trials, and suggests that completion lymph node dissection may be safely omitted in patients who respond well to neoadjuvant therapy.

## Data Availability

No datasets were generated or analysed during the current study.
